# “You can’t really have a relationship with them because they just ask you questions”: understanding adolescent dropout – an empirical single case study

**DOI:** 10.3389/fpsyg.2024.1381901

**Published:** 2024-03-12

**Authors:** Antonella Cirasola, Dora Szegedi, Peter Fonagy, Nick Midgley

**Affiliations:** ^1^Research Department of Clinical, Educational and Health Psychology, University College London, London, United Kingdom; ^2^Anna Freud National Centre for Children and Families, London, United Kingdom

**Keywords:** unsuccessful treatments, dropout, youth psychotherapy, alliance, Short-Term Psychoanalytic Psychotherapy, single case, adolescent depression

## Abstract

**Introduction:**

High dropout rates are common in youth psychotherapy, including psychoanalytic psychotherapy, yet the reasons behind this trend remain obscure. A critical focus to enhance adolescent engagement could be the therapeutic alliance, particularly in resolving alliance ruptures. This study sought to clarify the complex relationships between the therapeutic alliance, encompassing alliance ruptures and resolutions, and dropout within the context of poor outcome. It investigated a single case of an adolescent with depression who dropped-out of Short-Term Psychoanalytic Psychotherapy, without showing clinical improvement.

**Method:**

Data was garnered from diverse sources, including questionnaires, interviews, and session recordings, and analyzed through a mixed-method longitudinal framework. This encompassed views from the adolescent, therapist, parents, and external evaluators.

**Results:**

The study identifies several factors impacting the decision to drop out, including initial profound distrust toward the therapist, a complex and difficult therapeutic relationship characterized by unresolved alliance ruptures, and sporadic attendance. External factors including minimal parental engagement with therapy were also seen as detrimental to the adolescent’s involvement and progress.

**Discussion:**

The research underscores the challenges in engaging adolescents, especially when there may be distrust of professionals, and in the absence of parental involvement with treatment.

## 1 Introduction

Dropout from psychological treatment is a pressing issue in mental health services and is acknowledged as a significant challenge for clinicians (Leibovich et al., 2019), particularly when working with young people. Defined as a client discontinuing therapy without their therapist’s agreement, dropout is estimated to impact around 45% of young people commencing therapy (De Haan et al., 2013). It is often linked to dissatisfaction with the treatment (O’Keeffe et al., 2019b), and those who drop out may not fully reap the benefits of the treatment (Cooper et al., 2018), underlining the importance of understanding what causes dropout and how it can be avoided.

A number of factors have been examined in previous research as contributing to treatment dropout. While variables such as symptom severity, socioeconomic status, and ethnicity show inconsistent predictive power regarding dropout in youth therapy (O’Keeffe et al., 2018), the establishment of a strong therapeutic alliance early in treatment consistently appears as a significant factor when trying to prevent dropout. The alliance, as conceptualized within therapeutic contexts, pertains to the collaborative facets of the relationship between the patient and therapist, facilitating their joint efforts toward shared goals and the cultivation of a positive rapport (Bordin, 1979). A strong alliance has been found to be consistently associated with increased engagement and positive treatment outcomes across various treatment types for adolescents (Daly et al., 2010; Gersh et al., 2017; Schenk et al., 2019; Cirasola et al., 2022). On the contrary, poor alliance and unresolved issues within the therapeutic relationship, including alliance ruptures, have been found to be significantly linked with reduced engagement and a higher likelihood of dropout (Eubanks et al., 2018; O’Keeffe et al., 2020).

Alliance ruptures refer to difficulties in working together toward therapy goals and breakdowns in the therapeutic bond (Safran and Muran, 2000; Muran and Eubanks, 2020). These ruptures manifest through withdrawal or confrontation markers. Withdrawal rupture markers can be considered as ‘movements away’ from the therapist and/or the therapy and arise when clients either distance themselves from the therapist or the therapeutic process, for instance, by giving minimal responses, evasive storytelling, or exhibiting self-criticism and hopelessness. They may also involve moving toward the therapist, but in a way that disguises and distances them from their true feelings, such as through denial, separating content from emotion, or deferential behaviors. Conversely, confrontation markers can be considered as ‘movements against’ the therapist and/or the therapy and involve actions against the therapeutic work, including complaints, criticism, resistance, or attempts to control the session. A rupture is deemed resolved when the client and therapist re-establish a positive emotional connection and resume collaborative therapy.

Effectively addressing these relational issues is particularly vital when working with adolescents, a group with high incidence of ruptures and dropout (Schenk et al., 2019; O’Keeffe et al., 2019a; Cirasola et al., 2022). The unique developmental challenges of adolescence, such as increased individuation, identity exploration, and emerging autonomy, significantly influence these dynamics (DiGiuseppe et al., 1996; Binder et al., 2008; Schenk et al., 2019; Cirasola et al., 2022). Adolescence is marked by a push-pull between seeking autonomy and needing support and guidance, often leading to difficulties in forming and sustaining a therapeutic alliance with an adult clinician. Additionally, factors like evolving self-perception, changing interpersonal relationships, risk-taking behaviors, and cognitive development add to the complexities of engaging adolescents in therapy (Gulliver et al., 2010; Schenk et al., 2019; Cirasola and Midgley, 2023).

Understanding and effectively managing alliance ruptures is vital for successful psychotherapy, but research in this area has predominantly centered on adults, leaving a considerable knowledge gap in youth psychotherapy. Building a robust alliance and resolving ruptures is especially pertinent in psychodynamic treatments with young people. Empirical studies have identified frequent alliance ruptures in this context (Halfon et al., 2019; Schenk et al., 2019; Cirasola et al., 2022) and lower alliance ratings compared to other therapeutic modalities (Cirasola et al., 2021). This could be attributed to the psychoanalytic approach’s emphasis on creating a space where negative emotions, often manifested through negative transference, are openly expressed (Cregeen et al., 2017). Therapists in this framework strive to recognize and support the expression of these negative emotions toward the therapist, showing tolerance and acceptance. The deliberate encouragement of expressing negative emotions, along with other aspects of psychoanalysis that intentionally induce some frustration (e.g., not directly answering personal questions), may lead to more pronounced alliance ruptures, reflected in lower alliance ratings. The impact of this on treatment outcomes remains a subject for further investigation.

In psychoanalytic therapy, it is also critical to consider that certain techniques might challenge the therapeutic relationship. Unlike some other therapies, psychoanalytic psychotherapy often adopts a less directive approach, emphasizing the young person’s autonomy in exploring their own thoughts and emotions (Cregeen et al., 2017). This means therapists typically refrain from structuring sessions rigidly and limit self-disclosure, adopting a more ‘neutral’ stance to facilitate the young person’s exploration of their internal world, including transference. This approach can lead to prolonged silences, where therapists encourage young people to uncover their thoughts and feelings independently rather than providing immediate explanations or reassurance. While these techniques are fundamental to the psychoanalytic method, they may pose challenges for young people unaccustomed to this style of therapy. Research suggests that extended periods of silence, particularly when a young person is withdrawing or having difficulty expressing emotions, can adversely affect the therapeutic relationship (Acheson et al., 2020). Further research is needed to guide therapists in applying these techniques effectively, fostering a strong therapeutic relationship and addressing potential ruptures.

To address this need, this study utilizes a case study approach to investigate the complex relationships between therapeutic alliance, dropout rates, and unfavorable outcomes in adolescents with depression undergoing Short-Term Psychodynamic Psychotherapy (STPP). Specifically, this study aims to:

- Describe the development of the therapeutic alliance, including its ruptures and resolutions, within the context of a single STPP case involving an adolescent with depression who dropped out of therapy, and exhibited a poor treatment outcome.
- Explore the factors influencing the young person’s decision to discontinue treatment, with a specific focus on the role of the therapeutic relationship, particularly the alliance and its rupture and resolutions.

The case study design was selected for its ability to offer a detailed and context-rich examination of the intricate dynamics in patient-therapist interactions, providing insights that are not readily obtained through group-based analyses (McLeod, 2013). This focused methodology allows for an in-depth investigation, illuminating the complexities of the therapeutic relationship and its influence on treatment outcomes.

## 2 Materials and methods

### 2.1 Design

This study adopted a longitudinal, mixed-methods case study approach, examining a single case from the IMPACT-ME study (Midgley et al., 2014), the qualitative component of the IMPACT clinical trial, evaluating the effectiveness of three psychological treatments of depression in adolescents (Goodyer et al., 2017). Comprehensive details of the methods and procedures of the clinical trial and associated qualitative investigation are detailed in Goodyer et al. (2017) and Midgley et al. (2014).

### 2.2 Ethical considerations

The research protocol was approved by the Cambridgeshire 2 Research Ethics Committee (REC Reference: 09/HO308/137). Informed written consent was obtained from all participants, including parental consent. To maintain confidentiality, all identifying personal information has been anonymised.

### 2.3 Case selection

To select a suitable case, the IMPACT-ME database was examined to identify:

- Cases referred for Short-Term Psychoanalytic Psychotherapy (STPP);
- With available post-therapy interviews with the adolescent, their caregiver, and the therapist, to provide a comprehensive exploration of their views on the therapeutic relationship, process, and outcome;
- Recorded as ‘drop out’ in the study database, indicating therapy was ended prematurely without therapist agreement;
- Negative outcomes, indicated by scores consistently above the clinical threshold (27 or above) on the primary outcome measure, the Moods and Feelings Questionnaire (MFQ), at both the end of therapy and the one-year follow-up;
- With complete audio recordings of therapy sessions, to allow a thorough and comprehensive evaluation of changes in the therapeutic alliance;

Of the 27 STPP cases in the IMPACT-ME study, 18 had complete interviews. Among these, 9 were identified as dropouts, and of these 5 had poor outcome, but only the chosen case had the full set of sessions audio recordings. Further details of the case are provided, below.

In respecting the privacy of the individuals involved, certain specifics about the backgrounds of the young person and their family have been omitted or changed. However, all pertinent information relevant to the therapeutic alliance and its dynamics remains unaltered.

### 2.4 Treatment

Short-Term Psychodynamic Psychotherapy (STPP) as described by Cregeen et al. (2017), is a structured therapy consisting of up to 28 sessions over a period of 30 weeks. Parents were also given the opportunity to engage in up to seven sessions of parent work with a separate clinician. Based on psychoanalytic principles, STPP views behavioral and emotional issues as linked to early relational experiences and current developmental challenges. By actively engaging with both transference and countertransference, STPP seeks to reveal the deeper dynamics underlying symptoms, aiming to enhance adolescents’ ability to regulate emotions and develop and maintain healthy relationships.

The therapist offering the STPP, referred to here as Dr. P, was a male child and adolescent psychoanalytic psychotherapist, accredited by the Association of Child Psychotherapists in the UK. No further demographic information about the therapist was available.

### 2.5 Measures

#### 2.5.1 Alliance

The therapeutic alliance was assessed using the Working Alliance Inventory Short-form (WAI-S; Horvath and Greenberg, 1989; Tracey and Kokotovic, 1989) from both the adolescent and therapist perspectives at 6, 12, and 36 weeks post-randomization. The WAI-S comprises 12 items, rated on a 7-point Likert scale, measuring three dimensions: (1) goals agreement, (2) tasks agreement, and (3) emotional bond between client and therapist, with higher ratings indicating a stronger alliance. It demonstrates good construct validity (*r* = 0.74 to *r* = 0.80) and strong internal consistency in adult (*α* = 0.93) and youth samples (*α* = 0.94). In the IMPACT study also showed robust internal consistency (*α* = 0.95).

#### 2.5.2 Outcome

Aligned with the IMPACT study, this research used the 33-item MFQ (Angold et al., 1987) to assess self-reported depression symptoms. A score of 27 on the MFQ signifies the clinical threshold for a major depressive episode (Wood et al., 1995). Assessments occurred at baseline, and post-randomization at 6 and 12 weeks (during treatment), 36 weeks (end of treatment), and 52 and 86 weeks (post-treatment follow-ups). The MFQ demonstrates good reliability (*r* = 0.78), strong internal consistency (*α* = 0.82), and criterion validity (*α* = 0.89) for identifying adolescent depression episodes (Wood et al., 1995; Kent et al., 1997).

#### 2.5.3 Alliance rupture and resolutions

The latest version of the Rupture Resolution Rating System (3RS v2022; Eubanks et al., 2022) was used to identify alliance ruptures and resolutions. This observer-based measure codes ruptures and resolutions in recorded therapy sessions. Ruptures, categorized as withdrawal and confrontation, are assessed on a 5-point scale using the 3RS, with 1 indicating ‘Not present/salient’ and 5 indicating ‘Present, very salient.’ The salience reflects the impact on the alliance. Tables 1, 2 provide detailed descriptions of withdrawal and confrontation rupture markers, along with resolution strategies. Resolution effectiveness is indicated by repair anchors, ranging from 1 (Ruptures not successfully repaired, alliance worsened) to 5 (Ruptures well repaired).

**Table 1:** Description and mean salience of 3RS withdrawal and confrontation rupture markers.

Withdrawal			
Withdrawal markers	Description	Client mean salience (SD)	Therapist mean salience (SD)
Masks experience	*The person is being deferential/appeasing and withdraws from the work of therapy by exhibiting affect that does not match the content of their narrative.*	4.29 (0.76)	2.43 (0.98)
Avoids	*The person uses abstract communication, avoidant storytelling and topic shift to avoid the work of therapy.*	3.57 (0.53)	1.57 (0.53)
Shuts down	*The person withdraws from the work of therapy by being self-critical, denying, going silent or giving minimal responses to questions or statements that are intended to initiate or continue the discussion.*	3.00 (1.53)	1.86 (1.07)
Overall		4.29 (0.49)	2.14 (0.69)

Confrontation			
Confrontation markers	Description	Client mean salience (SD)	Therapist mean salience (SD)
Pushes back	*The person rejects the other one’s ideas, defends themself and/or is being hostile.*	3.29 (0.76)	1.86 (1.07)
Controls	*The person attempts to control the other one and/or the session or puts pressure on the other person to fix their problems quickly.*	2.86 (0.90)	2 (1.73)
Complains	*The person criticizes the other one, activities, interventions, parameters and/or progress.*	2.00 (0.82)	1.14 (0.38)
Overall		3.14 (0.69)	1.86 (1.21)

**Table 2:** Description and mean salience of 3RS resolution markers.

Resolution category	Resolution marker	Mean salience (SD)
Exploring the Rupture	*Therapist invites patients’ thoughts and feelings.*	3.71 (0.95)
	*Therapist validates the patient’s defensive posture.*	3.00 (0.53)
	*Therapist discloses their internal experience of the rupture.*	1.86 (1.07)
	*Therapist clarifies misunderstanding.*	1.43 (0.53)
	*Patient discloses or clarifies their internal experience of rupture.*	1.86 (1.21)
Linking to patterns	*Therapist links rupture to interpersonal patterns.*	2.71 (0.49)
Acknowledging contribution	*Therapist acknowledges their contribution to rupture.*	2.29 (1.25)
	*Patient acknowledges their contribution to rupture.*	1.42 (1.04)
Focusing on the task	*Therapist illustrates task/rationale.*	2 (0.82)
	*Therapist redirects back to task/work of therapy.*	1.86 (0.90)
	*Therapist proposes/discusses changing task/goal or changes task/goal.*	1.86 (0.90)
	*Patient discusses changing task/goal.*	1 (0.00)

The 3RS incorporates a Working Together (WT) subscale, based on Bordin’s (1979) alliance model. This subscale assesses goal agreement and task collaboration, trust, and contributions of the therapist and patient to their relationship on a 5-point scale. A score of 1, ‘Not salient,’ indicates ineffective collaboration, while 5, ‘Very salient,’ suggests a high level of collaborative effort. An average score of 3 indicates some degree of collaboration between the patient and therapist, possibly involving cooperative efforts or engagement in a shared task toward a common goal.

#### 2.5.4 Therapy interviews

Semi-structured interviews using the Experience of Therapy Interview (Midgley et al., 2011) were conducted at three time points: baseline (T1, before therapy started), 36 weeks post-randomization (T2, end of therapy), and 86 weeks post-randomization (T3, 1 year follow-up). An independent research psychologist conducted separate interviews with adolescents and their primary caregivers at all time points. Additionally, the therapist was interviewed at T2 only with the young person’s consent. These interviews aimed to capture diverse perspectives on the therapeutic process and outcomes from the therapist, patient, and caregiver.

### 2.6 Data analysis

To describe the alliance and its rupture and resolutions over the course of treatment, all session recordings were analyzed by two independent raters using the 3RS to evaluate the occurrence and type of alliance rupture resolutions. To achieve a comprehensive understanding of the therapeutic relationship and its influence on therapy outcomes and processes from multiple perspectives, we employed a dual-method approach. First, we used self-reported alliance ratings (WAI-S and WAI-T) obtained from both patients and therapists. Second, we conducted qualitative analyses of transcripts derived from seven interviews, conducted at different time-points, and involving the therapist, client, and caregiver. The interviews underwent independent analysis by two raters utilizing the framework analysis method, as outlined by Parkinson et al. (2016). Framework Analysis entailed the construction of an analytical framework, incorporating predefined research topics and emerging themes from the data (refer to Supplementary Table 1). Ultimately, the information gathered from these diverse sources was synthesized in a narrative form to comprehensively address the study’s aims using a multi-perspective approach.

#### 2.6.1 Epistemological position and reflexivity

This single case study employs a mixed-methods design from a critical realist epistemological perspective (McEvoy and Richards, 2006). Critical realism suggests that research data provide insights into reality but do not directly access it, thus necessitating a combination of various observations and analyses for a comprehensive understanding (Edgley et al., 2016). This approach underscores the importance of utilizing multiple measures and observations, which, despite potential errors, collectively enhance understanding and mitigate biases (Creamer and Reeping, 2020).

Given the variability of the alliance construct in youth psychotherapy, influenced by unique patient and therapist characteristics, therapy type, and dynamic interactions throughout treatment, this study employs a mixed method approached and various sources of information. These include questionnaires, interviews, and audio recordings of therapy sessions, gathered from various perspectives (e.g., adolescent, therapist, parent, observers).

From this epistemological position, we approached the data with a sense of curiosity and objectivity, seeking to mitigate the influence of subjective biases. To achieve this, not only did at least two independent researchers look at each set of data, but reflective accounts were diligently composed subsequent to the analysis of each therapy session and interview. Furthermore, multiple meetings were convened among the researchers and senior authors, fostering an environment conducive to the thorough exploration of the data. Despite the inherent potential for subjectivity in the analysis process, meticulous efforts were exerted to ensure the provision of a transparent and comprehensive portrayal of the case.

#### 2.6.2 Raters, inter-rater reliability and credibility checks

With regards to the sessions assessments, the first author, a qualified clinical psychologist, assessed each therapy session using the 3RS. To confirm the inter-rater reliability of these ratings, the second author independently re-evaluated sessions using the 3RS, but only after completing the interview analyses to minimize biases. Cohen’s linearly weighted kappa (κ) and the Intraclass Correlation Coefficient (ICC) were calculated to assess their level of agreement on the 3RS ratings. The results indicated excellent agreement on client withdrawal ruptures (*κ* = 1), substantial agreement on therapist withdrawal ruptures (*κ* = 0.81), rupture resolution (*κ* = 0.84), and the Working Together score (ICC = 0.81; using a two-way model with absolute agreement). Moderate agreement was observed for client confrontation ruptures (*κ* = 0.76) and therapist confrontation ruptures (*κ* = 0.71).

For the post-therapy interviews, the transcripts were independently examined by two post-graduate students, which included the second author and another independent researcher. Both were MSc psychology students and were blind to any information pertaining to the specific case. Subsequently, the different elements of the data underwent a comprehensive review and audit conducted by the first and last authors.

## 3 Results

### 3.1 Case overview

‘Morgan’ was 17 at the start of treatment, and was assessed as having severe depressive symptoms, including suicidal thoughts, sleep disturbances, and a downturn in academic performance. Due to the severity of her symptoms, alongside being referred to Child and Adolescent Mental Health Services (CAMHS), she was prescribed SSRI medication. As part of the IMPACT study, she was randomly assigned to attend Short-Term Psychodynamic Psychotherapy (STPP).

In the interviews, both the therapist and Morgan’s parents expressed serious concern about Morgan’s condition at the time of referral. Although Morgan expressed willingness to be “sorted out,” she also revealed hesitations and mistrust toward mental health professionals. Out of 28 planned sessions, Morgan attended only seven, and then dropped out of treatment after 15 weeks without formal agreement or communication with her therapist.

As displayed in the last column of Table 3, Morgan exhibited a high MFQ score at baseline, signifying severe depression. Although the score initially decreased after 12 weeks, it later increased and consistently remained within the clinical range at all time points. Therefore, Morgan aligns with the profile of a ‘halted improver’– essentially a non-responder to treatment, mirroring a pattern observed in approximately 15% of cases in the IMPACT study (Davies et al., 2019). In line with the persistently high MFQ scores, Morgan, her parents, and Dr. P. were all in agreement that Morgan did not experience significant benefits from the therapy. Yet, Dr. P. harbored the hope that through therapy, Morgan, at the very least, had a positive experience where someone actively advocated for her well-being.

**Table 3:** MFQ, WAI-S and WAI-S-T ratings at all time points.

Time-points	WAI-S	WAI-S-T	MFQ
Baseline	N/A	N/A	49
6 weeks	61	50	44
12 weeks	56	42	31
36 weeks	52	*	39
52 weeks	N/A	N/A	54
86 weeks	N/A	N/A	44

*Missing data.

### 3.2 The alliance and its dynamics in this case

As shown in the first two columns of Table 3, both Morgan (WAI-S) and Dr. P. (WAI-S-T) consistently rated the alliance just above average across all assessment points, with a gradual decline over time, which suggests a potential weakening of the alliance. While Table 3 indicates a decline in the alliance over time and a lack of improvement in symptoms, this pattern cannot in itself establish a direct causal link between a worsening in the alliance and therapeutic outcomes.

The challenges to developing and maintaining a good therapeutic alliance, as demonstrated by the WAI ratings, was further supported by the session-by-session 3RS ratings. Table 4 presents the ratings for 3RS Salience Ratings for the observed rupture and resolutions events, including overall resolution and working together scores, for each session. Furthermore, the last row displays the average salience scores for alliance ruptures, resolutions, and collaborative work (3RS) across all sessions. In alignment with the self-reported alliance ratings, the observer-based 3RS ‘Working Together’ scale indicates an overall average level of collaboration, with subtle decline in the later sessions compared to the initial ones, signifying a diminishing ability for Morgan and Dr. P. to work together and trust each other.

**Table 4:** 3 RS salience ratings scores for each session and mean (M) and standard deviation (SD) scores across all sessions.

Weeks	Session	Withdrawal		Confrontation		Overall resolution	Working together
		Client	Therapist	Client	Therapist		
1	1	4	2	3	1	2	2.6
2	2	4	2	3	2	3	3.2
3	3	4	1	2	1	4	3
4	4	4	2	3	1	3	3.2
5–7	DNA						
8	5	5	3	4	2	1	2.8
9	6	4	3	4	1	2	2.4
10–14	DNA						
15	7	5	2	3	2	2	2.6
M (SD) across sessions		4.29 (0.49)	2.10 (0.69)	3.16 (0.69)	1.70 (1.21)	2.43 (0.98)	2.83 (0.31)

DNA = Did Not Attend’, indicating that the young person did not attend the session and did not provide prior notification to the therapist.

Morgan’s consistent display of rupture markers and limited engagement throughout the treatment, as demonstrated by the 3RS scoring of the sessions, serves to support the identified patterns. Over the course of seven attended sessions, Morgan showed a large number of rupture markers, averaging 22 markers per session. Notably, withdrawal emerged as the predominant type, constituting 66% of the total rupture markers observed. These withdrawal markers had a notably higher impact on the therapeutic alliance compared to confrontational markers, as evidenced by ratings of 4 or above on the 3RS rupture salience scale across sessions.

Although confrontation rupture markers were less prevalent, they still exerted a moderate to high impact on the therapeutic alliance, particularly in later sessions, where they were rated 3 or above on the 3RS rupture salience scale. This observation aligns with the self-ratings of the alliance provided by both Morgan and Dr. P., confirming a potential deterioration in the alliance and an escalation of tension in the therapeutic relationship as therapy progressed, ultimately leading to dropout.

Furthermore, an analysis of the 3RS ratings of the resolution of the ruptures within and across sessions reveals that approximately 58% of the sessions scored 2 or below on the overall resolution scale, indicating suboptimal resolution of ruptures in the majority of sessions. This is particularly evident in sessions preceding dropout, where most ruptures remained unresolved.

Table 1 outlines rupture markers and their average impact in all sessions. Morgan’s most salient withdrawal rupture marker, ‘mask her real experience,’ reflected her tendency to disengage by expressing incongruent emotions, often through sarcasm, especially in challenging situations. An example of this is illustrated in the following session excerpt, where the corresponding 3RS markers are noted in parentheses:

Therapist (T): I’ve noticed that— this is going to sound like a criticism— but I’ve noticed that you do not have a routine, that you do not sort of manage your own life in a sense.

Morgan (M): Nope! (loud and funny tone) [mask her real experience].

T: No. And that’s a bit, like, It’s a bit like you are kind of floating about like a bit of a mess sometimes.

M: (laughs) I am like a cloud! (funny, loud tone) [content-affect split].

Morgan also frequently showed indications of withdrawal ruptures in therapy by (a) evading through actions like switching topics, going off on tangents, and using abstract language, or (b) shutting down with minimal responses and inadequate engagement in discussions. An example is provided below, where Morgan used vague language when asked about her feelings toward a friend:

T: You seem to be very close to her, how is she?

M: She’s how you’d imagine her to be [abstract communication & minimal response].

T: How is that? [invites thoughts and feelings].

M: Its (long pause) [minimal response].

T: People have all different types of friends, do not they? [invites thoughts and feelings].

M: Like (pause) nice and (pause) friendly (pause) (laughs) [abstract communication].

Although less frequent than withdrawal markers, Morgan regularly exhibited confrontation rupture markers too, which were rated as moderately affecting the therapeutic alliance, as indicated by 3RS salience ratings (see Tables 1, 4). The most salient confrontation marker involved Morgan pushing back against therapy or the therapist’s interventions. An example is provided below, where Morgan reacted to the therapist’s remarks with a mix of disbelief and humor:

T: So, you are sort of being critical of yourself for that… Is it like a voice in your head, like your thoughts telling you that you are--.

M: What a peculiar way of putting it! (loudly with an exaggerated tone, then chuckles) [rejects intervention].

T: (laughs) Is it? [Invites thoughts and feelings].

M: Yeah! (continues laughing) A voice in my head?? Yes, there is a voice in my head! I’ve gone mental! (Loudly, with a funny tone) [masks real experience, indirect complaints].

Morgan also demonstrated confrontation rupture markers when attempting to exert pressure or control over the therapist, particularly when asking him personal questions about his age or his work. These instances persisted despite the therapist’s efforts to steer the conversation back to other topics. Moreover, there were moments when Morgan expressed dissatisfaction with the therapist, indirectly indicating her preference for a female psychiatrist she had previously consulted.

Despite the therapist’s ongoing efforts to repair ruptures (averaging 21.4 attempts per session), their effectiveness was generally limited, with most sessions scoring 2 or below on the 3RS overall resolution scale, indicating a lack of resolution. Not only were Dr. P.’s efforts to repair ruptures mostly rated as unsuccessful, but he also displayed withdrawal rupture markers on some occasions (an average of 5.7 per session), even if these markers had minimal impact on the alliance (scoring below 2 on the 3RS salience score). These primarily involved ‘masking his real experience.’ For example, there were times when Dr. P.’s emotional responses seemed misaligned with the content being discussed, such as laughing when he appeared uncomfortable. This was most noticeable in response to Morgan’s personal questions. Dr. P. typically adhered to what might be considered a traditional psychoanalytic stance, avoiding direct personal disclosure (e.g., by replying ‘this is more about you than about me’ when asked a personal question), which was sometimes rated on the 3RS as a movement away from the young person (withdrawal rupture). Alternatively, he focused on exploring the underlying motives behind Morgan questions instead of directly answering them. Both approaches often resulted in increased withdrawal markers from Morgan, as shown in the following excerpt following Morgan’s query about the therapist’s life at her age:

T: Maybe you are wondering if erm I was like you at your age?

M: Not really, just trying to make conversation. [long silence] [rejects intervention, minimal response].

Table 2 presents a summary of Dr. P’s strategies for addressing ruptures, including their average significance. He mainly used exploratory strategies, which involved delving into the rupture and its underlying reasons or patterns. This typically included encouraging the young person to explore her thoughts and feelings and validating her experiences. For example, when Morgan discussed upsetting issues with humor, the therapist promoted further exploration (e.g., ‘Oh gosh, what’s it like for you?’). In situations involving confrontational ruptures, such as Morgan expressing dissatisfaction with an intervention made by Dr. P., he responded by acknowledging and validating Morgan’s feelings, regardless of their negativity, as in the following example where he addressed Morgan’s challenges in engaging with therapy:

T: I get that, therapy is a really strange process to get used to, is not it? […] And it takes a bit of getting used to it because normally you know, you do not usually say everything that’s in your mind, do you? [validate defense].

Dr. P. also used immediate resolution strategies aimed at steering the therapy back on course or reducing the emotional intensity. For instance, when Morgan deviated from a topic in an avoidant manner, Dr. P. redirected the conversation to more pertinent subjects. Moreover, when facing signs of Morgan’s withdrawal ruptures, such as minimal responses, he showed flexibility by shifting to a different topic, as demonstrated in the following exchange:

T: Hmm. Probably more importantly, how are you feeling? (both laugh).

M: Yeah. (quietly) I’m okay. (long silence) [minimal response].

T: So, painting is something that you have always done for pleasure, is not it? [change topic].

Despite the therapist’s continued efforts to address ruptures, few ruptures were successfully repaired. Of these, a notable instance of rupture resolution occurred when the therapist authentically shared his feelings. This not only repaired the rupture but also actively involved Morgan in the resolution process.

T: — And I noticed that it was, um, it was after I told you that we were going to have a break over the holiday, that you did not come. [invites thoughts and feelings].

M: Yeeeaah aaaand???? (exaggerated, loud tone) [controls/puts pressure on the T].

T: Um, I was thinking about what you were saying about feeling abandoned and I was wondering if sometimes, without thinking, that you wanted to give me an experience of being abandoned. [invites thoughts and feelings].

M: Nooo! (exaggerated, loud tone) [pushes back/rejects idea].

T: Not consciously […] and I might not have explained myself correctly [acknowledge contribution].

M: Yeah! I wasn’t trying to make you feel abandoned! [rejects intervention].

T: No, I’m not saying it was like “right! I’m going to” [clarify misunderstanding].

M: “I’m going to abandon him!” (Loudly, with an exaggerated tone).

T: […] I just mean sometimes people do things without thinking, and it’s kind of giving me the personal experience of what it’s like [clarify misunderstanding]. And I wonder…

M: Did you feel abandoned? (Exaggerated tone) [puts some pressure on the therapist].

T: I felt concerned about where you were and… um… worried and… yeah I suppose, maybe abandoned is not quite the right word, but yeah, it certainly was an experience of not knowing what was going on [disclose internal experience].

M: Hm. Sorry for not turning up on those days. It wasn’t intentional. [P acknowledges contribution].

Despite the therapist’s persistent efforts, such as in the extract above, effectively engaging Morgan proved to be a persistent challenge as evidenced by her repeated session absences and sustained withdrawal rupture behaviors that remained unresolved. The ratings on the 3RS align with findings obtained from qualitative analysis of interviews conducted with both Morgan and the therapist. As elaborated below, both parties characterized their relationship as inherently challenging, offering insightful perspectives on the factors contributing to the intricacies of their interaction and how these dynamics may have impacted the overall therapeutic outcome.

### 3.3 Therapeutic relationship and treatment drop-out

Morgan’s decision to end the therapy prematurely without informing Dr. P. seems to be consistent with her tendency to withdraw when faced by alliance ruptures, as emerged from the session ratings. In her interview, she explained her decision to end therapy without first speaking to the therapist, stating: “It would have made me feel really guilty, so I just did not go.” Various factors influenced this decision, encompassing, but not limited to, a challenging relationship with Dr. P.

In the post-therapy interviews, Morgan identified a number of issues that contributed to her decision to drop out of therapy, including: (a) perceiving the therapy as unhelpful, (b) feeling inherent mistrust of mental health professionals, and (c) challenges in her relationship with Dr. P. From the therapist’s perspective, potential contributors to the decision were (a) Morgan’s difficulty in making use of the offered interventions, (b) challenges in their relationship, and (c) external circumstances in Morgan’s life, especially the lack of parental involvement in the therapy. The parents’ interviews did not uncover any additional factors. Morgan’s parents mainly mentioned the lack of progress and appeared to have limited knowledge about Morgan’s therapy experiences. They only shared Morgan’s reason for discontinuing therapy when she expressed, ‘It’s making me feel terrible. I do not want to continue.’ Moreover, the parents stressed that they were dealing with family issues, which hindered their ability to engage actively in Morgan’s treatment and they did not attend any of the parent sessions offered to them.

A key impediment to treatment engagement appeared to be Morgan’s skepticism toward mental health professionals. She openly conveyed her belief that clinicians, including Dr. P., could not fully understand or genuinely care about her difficulties. She acknowledged how this created a barrier from the onset of therapy, stating that her difficulty in trusting people “probably pre-determined that [she] wasn’t gonna open up.” This sense of distrust persisted over time, evident in her sentiments during the one-year follow-up interview: ‘I do not know…what they [mental health professionals] do but it just they make you feel so small and insignificant…(sighs)… (pause)… acting like they care.” This underscores how her initial reluctance to trust the therapist remained a persistent issue even after the therapy had ended.

Both Morgan and Dr. P. characterized the therapeutic relationship as challenging, and this dynamic appeared to have influenced the lack of engagement. While both parties acknowledged challenges in their relationship and found common ground on some contributing factors, they also highlighted unique elements that played a role in exacerbating these difficulties.

Morgan expressed dissatisfaction with the therapeutic relationship, particularly noting what she felt was its one-sidedness. She emphasized the lack of personal disclosure from the therapist, hindering the formation of a meaningful connection. She even said that she was not able to provide an in-depth description of their relationship, since she felt: “you cannot really have a relationship with them because they just ask you questions… but I wanna know what they are like as a person.” According to her, what impeded the formation of a meaningful relationship was the lack of personal disclosure from the therapist. Morgan conveyed a wish for a more “friendly” and “open” relationship with Dr. P. She desired open conversation and responsiveness to her inquiries instead of feeling like she was being “interrogated.” She reflected that her need to know more about the therapist possibly stemmed from her trust issues: “I have awful trust issues, so to talk to someone I actually have to… sort of know what they are like… personally….” The perceived lack of personal sharing from the therapist made the therapeutic interaction seem impersonal, making it “harder [for her] to talk about [her] feelings with Dr. P.”

Dr. P. also described the relationship with Morgan as challenging, marked by difficulties in engaging her and an awkward dynamic. He noted challenges arising from Morgan’s personal questions and his reluctance to disclose personal information. He talked about the difficulties he experienced in managing Morgan’s ‘intrusive’ questions while trying to establish a therapeutic connection. He explained that he struggled to find a balance between avoiding counterproductive dynamics while being sensitive to ‘her very strong sense of rejection.’ This complex interplay added to the challenges of their therapeutic relationship, which already suffered from a lack of engagement. Despite his continues efforts, Dr. P. acknowledged that their relationship did not get easier, and in his interview he consistently described their interaction as ‘awkward’ and ‘uncomfortable.’

Morgan’s fear of abandonment also emerged as a factor hindering the establishment of a positive and strong relationship with Dr. P., as acknowledged by both parties. In her interview, Morgan explicitly stated, “Dr. P. is gonna fucking abandon me too,” expressing the belief that opening up to him would be pointless. This fear intensified with the awareness of the time-limited treatment, causing disappointment when reminded in the sessions by Dr. P. of the “amount of weeks left.” Additionally, Morgan’s sporadic attendance, frequently addressed by the therapist, became a sensitive issue triggering guilt in her and reducing motivation, ultimately contributing to ruptures in the therapeutic alliance.

Dr. P. also acknowledged the impact of Morgan’s fear of abandonment on their relationship, especially during therapy breaks. Accordingly, he said that he tried to approach these breaks with sensitivity, recognizing their significance. However, he recognized that addressing Morgan’s poor attendance while avoiding making her feel criticized, became a delicate matter. He reflected on a particular session where his response to Morgan’s non-attendance at the previous session, his words might have been perceived as criticism by Morgan, potentially exacerbating the challenges in her therapy creating tension (or ruptures) in their relationship and potentially contributing to her decision not to continue with therapy. Notably, Morgan also acknowledged the difficulty posed by therapy breaks, impacting her motivation to continue the sessions. In fact, her decision to end therapy followed an extended break, as she reflected, ‘basically the break was about 3 weeks… and after that, I did not see the point in going anymore.’

In addressing the challenges encountered in establishing a meaningful therapeutic alliance with Morgan, Dr. P. emphasized the detrimental impact of her poor attendance, unresponsiveness to messages, and difficulty in ‘engaging with the session structure.’ Dr. P., while perceiving that Morgan, to a certain extent, appreciated his efforts to connect, described what he saw as her inability “to use what was offered to her.”

According to Dr. P., external factors also significantly impacted Morgan’s ultimate decision to stop going to therapy, underscoring common complexities in adolescent complex cases. These factors included Morgan’s difficulties at college, and especially, the absence of parental involvement in the therapy. Dr. P. clarified that Morgan’s parents neither attended any offered sessions nor responded to the therapist’s attempts to communicate or gather information about Morgan’s attendance issues. According to Dr. P., this lack of parental engagement played a crucial role in hindering Morgan’s involvement in therapy. These factors collectively created substantial barriers to consistent therapy attendance, and as Dr. P. concluded: “It was unrealistic to expect her to be able to get to therapy.”

## 4 Discussion

This case study offers an in-depth analysis of the therapeutic relationship and the factors contributing to a decision to drop out of psychoanalytic psychotherapy with Morgan, an adolescent who was experiencing depression. The study highlights the complexities inherent in engaging an adolescent in therapy.

The exploration of the relationship between Morgan and Dr. P. revealed several issues and a considerable number of alliance ruptures, predominantly of the withdrawal type. These ruptures were marked by Morgan’s tendency to disengage from the therapeutic process, manifesting in behaviors such as avoidance, giving minimal responses, and communicating in abstract terms. The prevalence of withdrawal over confrontation ruptures aligns with findings from previous research in youth psychotherapy (Gersh et al., 2017; Schenk et al., 2019; O’Keeffe et al., 2019b; Cirasola et al., 2023). This pattern suggests that withdrawal ruptures may be more characteristic of youth populations, who may find it challenging to engage in therapy and are often more inclined toward withdrawal behaviors (Johnson et al., 2009; Constantino et al., 2010).

The majority of the withdrawal ruptures in Morgan’s case persisted without resolution, which may have been a significant factor in the difficulties encountered in establishing a robust therapeutic alliance. This unresolved state likely contributed to Morgan’s decision to stop going to therapy. This observation is in line with existing research suggesting that unresolved ruptures can lead to poorer engagement and treatment outcomes (Eubanks et al., 2018). Additionally, these findings resonate with other studies highlighting that adolescents, when dissatisfied with therapy or the therapeutic relationship, tend to withdraw and drop out of treatment, often without explicitly expressing their dissatisfaction to the therapist (O’Keeffe et al., 2019a, 2020).

Our findings align with research in adult therapy, suggesting that both therapists and clients in unsuccessful cases tend to report less positive client-therapist relationships and therapy experiences compared to successful cases (Gazzillo et al., 2014; Hayes et al., 2015; Werbart et al., 2019a). However, our results diverge somewhat from another study conducted with therapists of non-improved young adults. That study showed these therapists described therapy outcomes favorably, noting increased insight and mitigated problems, yet they also reported an incoherent, split picture of the therapeutic process (Werbart et al., 2019b). In our case, the therapist demonstrated awareness of difficulties in the therapeutic relationship and process, as well as the lack of patients’ improvement. Since even the most skilled therapists can encounter unsuccessful treatments—patients who do not improve— further research is needed to assess the therapist’s impact on the therapeutic process in both successful and unsuccessful cases to understand what makes a difference. This is particularly relevant in youth therapy, given the challenges in engaging young people and their high dropout rates.

The results of this study also emphasize the importance of trust in the therapeutic process. Epistemic mistrust refers to a deep-seated skepticism or lack of confidence in the knowledge, intentions, or expertise of others, particularly in the context of mental health professionals and therapeutic relationships (Fonagy and Allison, 2014). Morgan’s epistemic mistrust, evident in her overt expressions of distrust toward mental health professionals, presented a significant barrier to her engagement from the onset of therapy. In psychotherapy, individuals with epistemic mistrust may be reluctant to disclose personal information, question the competence of their therapist, or harbor doubts about the effectiveness of the therapeutic process. Addressing and understanding epistemic mistrust is vital in mental health care, as it profoundly affects the dynamics of the therapeutic relationship and can significantly hinder successful treatment outcomes (Fonagy et al., 2015).

Morgan’s struggles with relating to and trusting the therapist shed light on her underlying issues and internal world. For example, her frequent withdrawal ruptures and distrust may suggest difficulties in social interaction, possibly stemming from negative or ambivalent attitudes toward self and others. Hence, ruptures can be viewed as coping mechanisms in response to the tension between two fundamental human motivations: agency and relatedness (Blatt, 2008). Through an examination of these ruptures, therapists can glean insights into patients’ personality traits and internal struggles. Therefore, it might be helpful for therapists to identify and interpret ruptures as glimpses into their clients’ inner worlds. Addressing and resolving these ruptures offers an opportunity to address maladaptive relational patterns and find a balance between self-definition and relatedness.

It is challenging to identify precisely what might have fostered a greater sense of trust in Morgan’s therapeutic relationship and aided in resolving ruptures. However, her explicit remarks about Dr. P’s refusal to answer more personal questions may be significant. Morgan expressed desire to know more about her therapist as a person and the absence of such information from Dr. P. likely contributed to her feeling of being treated impersonally. In the context of this STPP case, the lack of therapist self-disclosure is not surprising, aligning with the historical standpoint in psychoanalysis where self-disclosure is actively discouraged. This skepticism arises from the fundamental principles of psychoanalysis, where therapeutic focus traditionally centers on the patient’s exploration of their unconscious mind and dynamics. Within this framework, therapists traditionally maintain a neutral and objective stance, refraining from personal revelations to prevent potential interference with the patient’s introspective process (Campos, 2020). Contemporary psychoanalytic perspectives challenge the historical stance on therapist self-disclosure, highlighting potential benefits such as promoting authenticity and aiding clients in overcoming impasses and resistance (Malan and Coughlin Della Selva, 2007; Campos, 2020). This is especially relevant when working with clients like Morgan, who display epistemic mistrust, resulting in negative expectations of the therapist. Whilst this may be taken up as ‘negative transference,’ there appears to be a risk that it can contribute to treatment dropout, when there is not a fundamental sense that the therapist is ‘on my side.’ Therefore, appropriate self-disclosure and responsiveness to personal questions can foster honesty and authenticity, offering information that proves instrumental in overcoming therapeutic impasses and resistance (Gorkin, 1987).

In contrast to the historical perspective in psychoanalysis, contemporary alliance literature recognizes the therapist’s disclosure of their internal experience in the patient-therapist interaction as a reparative strategy (Eubanks et al., 2015, 2019). When employed with sensitivity, this strategy is seen as a means to flexibly negotiate distance and closeness based on the patient’s needs, rather than rigidly adhering to a specific treatment manual. Specifically, it is argued that judicious and well-considered self-disclosure can enhance the therapeutic relationship by offering clients insight into the therapist’s humanity, thereby fostering a more genuine connection (Muran and Eubanks, 2020). This approach is also endorsed in mentalization-based therapies, where therapists are encouraged to engage in careful self-disclosure as a vital means for both client and therapist to understand each other by sharing their respective thinking and emotional processes in listening and reacting to each other (Bateman and Fonagy, 2004). Although further research is required on this subject, it appears that, especially for clients with trust issues and signs of withdrawal ruptures (i.e., movement away from the therapist or therapy), both of which are common among adolescents, therapists who disclose personal information may be perceived as initiating a “movement toward” the clients. This could enhance the therapist’s authenticity and approachability, possibly leading to increased trust and greater engagement in therapy.

Another factor that likely influenced the difficulty in establishing trust with Morgan was the brief and time-limited nature of the treatment. The predetermined number of sessions in short-term therapy might have unintentionally intensified Morgan’s existing trust issues. Her fear of abandonment, heightened by the knowledge that the treatment had a set endpoint, mirrors a common challenge in time-limited psychotherapy (Norcross and Wampold, 2018). The constraint of a fixed number of sessions can present particular challenges for clients with histories of abandonment or difficulties in establishing trust and more research is needed on the topic.

In addition to the inherent complexities of the therapeutic relationship, this case study underscores the crucial role of external factors in shaping engagement with psychotherapy. Morgan’s numerous challenges, coupled with the absence of parental support for treatment—both indirect (such as endorsing therapy) and direct involvement—seemed to create substantial additional obstacles in the therapy process, especially when Morgan herself was struggling to engage. This aligns with an expanding body of research that underscores the importance of establishing a parental alliance when working with adolescents, particularly in complex cases (Novick and Novick, 2013; Forsberg et al., 2014; Feder and Diamond, 2016; Malberg, 2021). This approach can be instrumental in navigating external challenges and highlights the criticality of not only addressing internal psychological factors, especially issues in the therapeutic relationship, but also engaging with and leveraging external support systems to provide a comprehensive and effective therapeutic approach for adolescents struggling with complex mental health needs.

### 4.1 Strengths and limitations

The study exhibits several notable strengths, primarily evident in its comprehensive mixed-methods design from a critical realist epistemological perspective. By employing multiple methods, including questionnaires, interviews, and audio recordings of therapy sessions, the study was able to gather rich and diverse data from multiple perspectives, enhancing the depth of understanding. Another strength lies in the engagement of multiple assessors for rating alliance rupture-resolution events and conducting post-therapy interviews. This multifaceted approach, coupled with the adoption of blind raters for the 3RS, enhances the methodological rigor of the investigation.

However, there are also certain limitations in the study. The focus on a singular case warrants caution regarding the generalizability of findings. While such an approach offers in-depth insight into the intricacies of a particular therapeutic dyad, it restricts the ability to generalize findings to broader populations or contexts. Each therapeutic relationship is unique, influenced by a multitude of factors such as individual personalities, therapeutic techniques, and external circumstances. Therefore, extrapolating conclusions from a solitary case may overlook the variability present in different therapeutic settings. Additionally, the subjective nature of qualitative analysis implies that interpretations may vary between researchers. However, to mitigate biases, this study employed multiple independent raters and utilized reflective practices and collaborative discussions across various raters. Future research should aim to replicate these findings across multiple cases to enhance their applicability.

### 4.2 Conclusion

In conclusion, this single-case study provides valuable insights into the nuanced dynamics contributing to dropout from short-term psychoanalytic therapy among depressed adolescents. It elucidates the intricate interaction between therapeutic processes and a variety of external and internal factors. It highlights the role of the therapeutic relationship, especially alliance ruptures, highlighting the significance of identifying and addressing ruptures while mitigating epistemic mistrust to enhance the likelihood of therapeutic engagement. Establishing an alliance and repairing ruptures necessitates a flexible negotiation of distance and closeness tailored to the patient’s needs, and some aspects of a psychoanalytic approach may need to be adapted accordingly. While the study’s results may bear the unique characteristics of this specific case, the lessons derived from it can be instructive for therapists working with other cases. Notably, strategic self-disclosure emerges as a promising avenue for fostering connection with adolescents exhibiting epistemic mistrust and its associated challenges. These insights advocate for a reconsideration of clinical methodologies and therapist training, tailored to better equip practitioners to navigate ruptures with adolescents, especially those experiencing poor engagement and epistemic mistrust.

## Data availability statement

The data analyzed in this study is subject to the following licenses/restrictions: the data comprises audio recordings of sessions, which cannot be shared due to ethical and confidentiality considerations. Further inquiries can be directed to the corresponding author.

## Ethics statement

The research protocol was approved by the Cambridgeshire 2 Research Ethics Committee (REC Reference: 09/HO308/137). Informed written consent was obtained from all participants, including parental consent. To maintain confidentiality, all identifying personal information has been anonymised. The studies were conducted in accordance with the local legislation and institutional requirements. Written informed consent for participation in this study was provided by the participants’ legal guardians/next of kin. Written informed consent was obtained from the individual(s) for the publication of any potentially identifiable images or data included in this article.

## Author contributions

AC: Conceptualization, Data curation, Formal analysis, Investigation, Methodology, Project administration, Resources, Software, Supervision, Validation, Visualization, Writing – original draft, Writing – review & editing. DS: Formal analysis, Writing – review & editing. PF: Supervision, Writing – review & editing. NM: Conceptualization, Methodology, Supervision, Writing – review & editing.
