# Supplementary material for: “You can’t really have a relationship with them because they just ask you questions”: understanding adolescent dropout – an empirical single case study
Source: Front Psychol. 2024 Mar 12;15:1381901. doi: 10.3389/fpsyg.2024.1381901 (PMC10963658; doi:10.3389/fpsyg.2024.1381901)
Supplement: Supplementary file 1 [file Table_1.DOCX]

Supplementary Material

**Table SI.** *Framework Used for the Qualitative Analysis of the Interviews.*

| **Framework** | **Positive** | **Negative** |
| --- | --- | --- |
| **Outcome:**  What patient, therapist and parent felt had changed. |  |  |
| **Therapeutic relationship:**  What was the patient, therapist and parent's view of the therapeutic relationship, including their first impression, how it evolved, and important elements/moments (both positive and negative) of their relationship? |  |  |
| **Therapeutic process and outcome:**  What patient, therapist and parent felt was significant in treatment with particular focus on the therapeutic relationship and contributed to the outcome. |  |  |
